# Functional Properties of Casein and Caseinate Produced by Electrodialysis with Bipolar Membrane Coupled to an Ultrafiltration Module

**DOI:** 10.3390/membranes12030270

**Published:** 2022-02-26

**Authors:** Rosie Deschênes Gagnon, Laurent Bazinet, Sergey Mikhaylin

**Affiliations:** 1 Institute of Nutrition and Functional Foods (INAF), Dairy Science and Technology Research Center (STELA), Food Science Department, Université Laval, Quebec City, QC G1V 0A6, Canada; rosie.deschenes-gagnon.1@ulaval.ca (R.D.G.); laurent.bazinet@fsaa.ulaval.ca (L.B.); 2 Laboratoire de Transformation Alimentaire et Procédés Électromembranaires (LTAPEM/Laboratory of Food Processing and ElectroMembrane Processes), Food Science Department, Université Laval, Quebec City, QC G1V 0A6, Canada; 3 Laboratory of Food Sustainability (EcoFoodLab), Food Science Department, Université Laval, Quebec City, QC G1V 0A6, Canada

**Keywords:** casein, caseinate, bipolar membrane electrodialysis, ultrafiltration, functional properties

## Abstract

Electrodialysis with a bipolar membrane coupled to an ultrafiltration module (EDBM-UF) is a hybrid technology recently developed as an ecofriendly alternative to chemical acidification to produce casein and caseinate from skim milk. In this study, the composition and functional properties of casein and caseinate obtained by chemical acidification/basification and by the EDBM-UF method from winter and summer milks were analyzed and compared. Results show that the emulsifying properties, solubility, water holding, and gelling capacities are equivalent between casein and caseinate from both methods. However, the foaming properties of EDBM-UF ingredients were improved, and casein was less hygroscopic. Additionally, the season of milk influenced certain functional properties, such as water-holding capacity and hygroscopicity. Therefore, these results allow concluding that EDBM-UF ingredients have equivalent or higher functionality than chemically produced ingredients, and that the EDBM-UF process would be a more eco-efficient alternative to the chemical one.

## 1. Introduction

In the food industry, casein has attracted wide interest due to its nutritional value and functional properties [1,2]. Indeed, this major milk protein is the dietary protein having excellent nutritional quality due to its ability to meet the needs of essential amino acids and high digestibility [3]. In addition, this protein is a great source of bioactive peptide precursors [4]. Due to these many attributes, casein and its derived ingredients are used as food additives in various food and non-food products. Sodium caseinate is the most commonly used casein-based ingredient [5]. This ingredient is a soluble form of casein obtained via the neutralization of acid casein by NaOH. This soluble form increases its potential applications as a food ingredient, notably in baking products, pastry, and dairy products [2].

Caseins are produced by several methods, such as microfiltration, ultracentrifugation, rennet coagulation, and the most commonly used method, precipitation by chemical acidification to the isoelectric point [6,7]. A new method coupling electrodialysis with bipolar membrane and an ultrafiltration unit (EDBM-UF) has recently been developed for casein and caseinate production as an alternative to chemical acidification [8]. In this hybrid technology, milk passes through an ultrafiltration membrane, which allows retaining milk proteins. The UF permeate, composed of lactose and minerals, circulates into the EDBM cell, and when an electrical current is applied, UF permeate is electroacidified by the H^+^ ions generated by the bipolar membrane. In addition, permeate is demineralized due to the migration of cations through the cation-exchange membrane. The acidified and demineralized permeate is used to reduce milk pH and precipitate casein [9]. Due to the low ionic strength resulting from permeate demineralization, casein precipitates at pH 5.0 instead of 4.6, in comparison with chemical acidification, which allows better retention of Mg^2+^ and Ca^2+^ ions in final casein [9]. For caseinate production by the EDBM-UF method, NaOH produced in the alkali section of the electrodialysis module is used [9]. Furthermore, the special design of EDBM and UF modules was developed to avoid scaling and fouling on the ion-exchange membranes. Indeed, UF allows retaining proteins, which prevents protein clogging inside the EDBM cell and at the interface of the bipolar membrane where H^+^ ions are generated. In addition, the particular stacking of ion-exchange membranes in the EDBM cell avoids scaling by separating the basification compartment, where OH^−^ ions are generated on the anionic side of the bipolar membrane, from the mineral concentrate compartment where scaling-forming ions of Ca^2+^ and Mg^2+^ are present [9]. This approach has many advantages in comparison to chemical acidification, such as the better extraction yield and higher purification rate of casein. Additionally, this method demonstrated a lower environmental impact than the chemical one [8]. Moreover, in the various studies that have been carried out to develop this process, milks from different seasons have also been used, resulting in different values of pH for casein precipitation, between 4.8 in summer [9] and 5.0 in winter [10]. However, the functional properties of the casein and caseinate produced from summer and winter milks by EDBM-UF have never been studied.

In this context, the aim of the present study was to compare the composition and functional properties such as solubility, water holding capacity, hygroscopicity, and gelling capacity as well as the foaming and emulsifying properties of casein and caseinate obtained by chemical acidification/basification and EDBM-UF methods. Additionally, the impact of milk seasonality was investigated.

## 2. Materials and Methods

### 2.1. Materials

Milk used to produce casein and caseinate was ultrafiltered skimmed milk Lactantia Pure Filtre; Lactalis, (Toronto, ON, Canada) bought in summer (June–July 2020) and winter (February–March 2021). The oil used for testing the emulsion properties was canola oil Mazola; CH Food Companies, Inc. (Oakbrook Terrace, IL, USA). The NaOH, HCl, NaCl, and H_2_SO_4_ were purchased from Thermo Fisher (Nepean, ON, Canada) and the HNO_3_ and the Glucono-Delta-Lactone were purchased from Anachemia Canada Inc. (Montreal, QC, Canada).

### 2.2. Protocol

#### 2.2.1. Production of Casein and Caseinate by EDBM-UF

**Figure 1:**
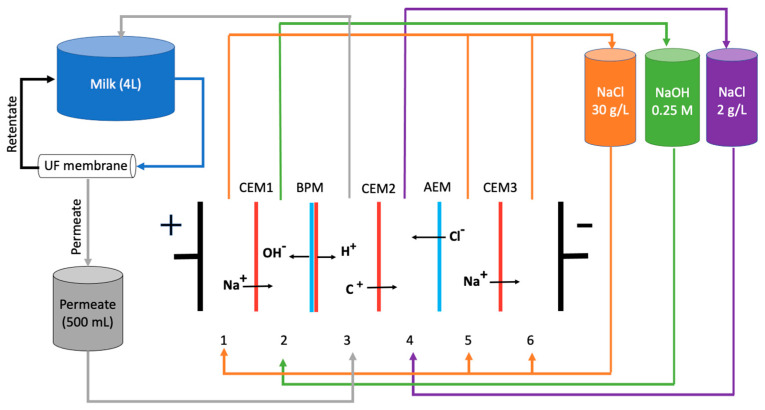
Configuration of the electrodialysis cell with bipolar membrane coupled to an ultrafiltration module (EDBM-UF). C^+^: migrating cations; CEM: cation exchange membrane; BPM: bipolar membrane; AEM: anion exchange membrane.

A volume of 4 L of skim milk was filtered at 30 psi through an UF module composed of a spiral-wound membrane with a filtration area of 2.14 m^2^ and 10 kDa molecular weight cut-off. The UF permeate, composed of water, minerals, and lactose, circulated inside the EDBM stack for acidification and demineralization (Figure 1). The EDBM module consisted of a bipolar membrane Neosepta BP-1, Astom, (Tokyo, Japan), three cation exchange membranes Neosepta CMX-SB, Astom, (Tokyo, Japan), and an anion exchange membrane Neosepta AMX-SB, Astom, (Tokyo, Japan), which formed six compartments. The electric current was maintained at 2 A throughout the process, according to the previous work of Masson et al. [9]. When the electrical current was applied, H^+^ and OH^−^ ions were generated by the bipolar membrane at the cation and anion exchange interfaces, respectively. Therefore, the UF permeate was acidified by the protons and demineralized via the migration of cations through the cation exchange membrane to compartment 4. The acidified and demineralized permeate was reintroduced into the milk reservoir for acidification and casein precipitation. A 0.25 M NaOH solution was circulated in compartment 2 where Na^+^ ions migrated from compartment 1 and OH^−^ ions were generated by the bipolar membrane. A 2 g/L NaCl solution was circulated in compartment 4 becoming concentrated in minerals, cations migrating from the permeate, and Cl^−^ ions from the 30 g/L NaCl solution.

To separate casein curd from whey after electroacidification, a centrifugation was carried out during 15 min at 11,000× *g* and 4 °C using an Avanti J26 XP centrifuge Beckman Coulter, (Brea, CA, USA). Then, supernatant was removed, and casein curd was washed with 1.8 L of distilled water by two successive centrifugations using the same centrifugation parameters. Then, washed casein curd was crushed into small particles using a food chopper KitchenAid, KFC3511OB (Mississauga, ON, Canada). For each type of milk (winter and summer), this process was carried out for 6 repetitions, from which 3 repetitions were used to produce casein and 3 repetitions were used to produce sodium caseinate.

Sodium caseinate was produced by the neutralization of casein curd using NaOH generated by EDBM in compartment 2. The concentration of electrogenerated NaOH was determined by titration, using HCl (0.5 M) and phenolphthalein indicator. To reduce viscosity during neutralization, the total solid concentration of casein was adjusted to 0.25% by adding 40 °C water, under stirring. This solution was heated up to 75 °C in a water bath Shaking Bath, VWR (Monroeville, PA, USA) under stirring [11]. Then, NaOH was added gradually, until pH 6.7 was reached. Both casein and caseinate were freeze-dried for the further analyses.

#### 2.2.2. Chemical Production of Casein and Caseinate

In a beaker under stirring, chemical acidification was carried out by the gradual addition of HCl 0.5 M to 4 L of preheated milk at 25 °C until pH 4.6 was reached. The volume of HCl added was recorded. Casein curd was washed and freeze-dried following the steps mentioned previously. Caseinate was produced by the neutralization of casein with NaOH (0.5 M) until pH 6.7, as explained in the above section. As for the EDBM-UF process, for both types of milk (winter and summer), 6 repetitions were performed, and half of them were used to produce caseinate.

### 2.3. Analyses

#### 2.3.1. Milk Composition

Milk protein, fat, lactose, solids, nonfat solids, and casein content were measured according to the AOAC method no. 972.16 [12], using a LactoScope Delta; PerkinElmer, (Waltham, MA, USA).

#### 2.3.2. Global System Resistance

The global system resistance (*R*, in Ω) was calculated according to Ohm’s law (Equation (1)) using the voltage (*U*, in V) and current intensity (*I*, in A). Current intensity was read on the power supply (60 V Multi Range DC Power Supply model 9110, BK Precision, Yorba Linda, CA, USA), and the voltage was measured by conductivity meter (Model 3100, k = 1 cm^−1^, Yellow Springs Instrument, Yellow Spring, OH, USA).

(1) $$R=\frac{U}{I}$$

#### 2.3.3. Milk pH

Milk pH was measured by a VWR Symphony pH-meter model SP20 Thermo Orion, (West Chester, PA, USA). Milk samples were collected at initial pH as well as at pH 6.2, 5.8, 5.4, 5.0, and 4.6 (for chemical acidification).

#### 2.3.4. Membranes Characterization

Membrane thickness and electrical conductivity were measured before and after 3 runs of EDBM according to the procedure described by Lemay et al. [13]. Membranes were soaked in 0.5 M NaCl solution for 30 min prior to the analysis. For thickness measurement, an electronic digital micrometer of 10 mm diameter flat contact point from the Marathon watch company LTD (Richmond Hill, ON, Canada) was used. Six measurements were taken at different locations on the membrane, and the average thickness was calculated.

The membrane electrical conductivity was calculated using the membrane thickness measurements and the electrical resistance obtained from the membrane conductance, which was the average conductance of six measurements at different locations on the membranes. A YSI conductivity meter model 3100 Yellow Springs Instrument Co. (Yellow Springs, OH, USA) equipped with a specially designed clip from the Laboratoire des Matériaux Echangeurs d’Ions (Université Paris XII, Créteil, Val de Marne, France) was used [13].

#### 2.3.5. Casein and Caseinate Composition

##### Moisture Content

Moisture content (in percent) was measured as described by Masson et al. [9]. The sample of 1 g was dried at 105 °C for 36 h to evaporate water in a vacuum oven Isotemp Vacuum Oven Model 280 A, Thermo Fisher Scientific, (Waltham, MA, USA) and then weighed. The moisture content was calculated by the following equation:

(2) $$\mathrm{Moisture}\;\mathrm{content}=\left(1-\frac{\mathrm{Sample}\;\mathrm{masse}\;\mathrm{after}\;\mathrm{drying}}{\mathrm{Sample}\;\mathrm{masse}\;\mathrm{before}\;\mathrm{drying}}\right)\times 100$$

##### Lactose Content

Lactose content (in percent) was analyzed by HPLC (Waters Corp., Milford, MA, USA). Samples (0.25 g) were solubilized in HPLC-grade water and treated with Biggs–Szijarto solution to precipitate protein [14]. Then, solutions were centrifuged at 5000× *g* for 5 min. The supernatant was diluted in HPLC-grade water and filtered with a 0.45 μm nylon filter CHROMSPEC Syringe Filter, Chromatographic Specialties (Brockville, ON, Canada). Then, liquid samples were injected in an ICSep-ION-300 column Tans-genomic, (Omaha, NE, USA). A refractive index detector Hitachi, (Foster City, CA, USA) was used to quantify lactose [14].

##### Protein Content

Protein content was determined using the Dumas combustion method, with a Micro Leco Truspec device Leco Corp. (Saint Joseph, MI, USA). A nitrogen conversion factor of 6.38 was used [15]. The analysis was carried out in triplicate, and the results were expressed in percent.

##### Ash Content

Ash content was determined according to AOAC method no. 945.46 [16] by weighing 1 g of sample in preweighed crucibles. The crucibles were placed in a furnace Lindberg/Blue M Moldatherm Box Furnaces, Thermo Fisher Scientific (Waltham, MA, USA) at 550 °C for 18 h. Then, they were cooled in a desiccator before being weighed. The ash content (in percent) was calculated using the following equation:

(3) $$\mathrm{Ash}\;\mathrm{content}=\left(1-\frac{\mathrm{Sample}\;\mathrm{mase}\;\mathrm{after}\;\mathrm{incineration}}{\mathrm{Sample}\;\mathrm{mass}\;\mathrm{before}\;\mathrm{incineration}}\right)\times 100$$

#### 2.3.6. Functional Properties of Casein and Caseinate

##### Solubility

The solubility of sodium caseinate only was determined according to Haque et al. with some modifications [17]. A 5% *w*/*v* caseinate solution (50 mL) was stirred with distilled water using a magnetic stirrer for 30 min at room temperature, and 40 mL of this solution were transferred into 50 mL centrifugation tubes. These were centrifuged at 1000× *g* and 20 °C for 10 min. Then, 5 g aliquots of the supernatant were placed into aluminum containers, dried at 105 °C for 24 h, and cooled in a desiccator to room temperature [17,18]. Solubility (in percent) was calculated according to the following equation:

(4) $$\mathrm{Solubility}\;=\frac{\mathrm{Protein}\;\mathrm{in}\;\mathrm{the}\;\mathrm{supernatant}}{\mathrm{Protein}\;\mathrm{in}\;\mathrm{the}\;\mathrm{solution}}\times 100$$

Protein concentration was determined using the Dumas method as described above.

##### Hygroscopicity

Hygroscopicity was determined with the method described by Ma et al. with some modifications [19]. Samples of casein and sodium caseinate (1 g) were poured in Petri dishes and placed at room temperature in a desiccator, which contained a Na_2_SO_4_ saturated solution. Samples were weighed after one week. Hygroscopicity was expressed as g of water absorbed per 100 g of dry solids.

##### Water-Holding Capacity

The water-holding capacity of casein only was determined according to a slightly modified version of the method described by Mirmoghtadaie et al. [20] by dispersing a sample of casein (1 g) with 10 mL of distilled water and placing it in 15 mL centrifuge tubes. The dispersions were vortexed for 1 min and left for 30 min. Then, they were centrifuged at 3000× *g* at room temperature for 10 min. The supernatant was removed, and the pellet was weighed into preweighed aluminum containers and dried in a vacuum oven Isotemp Vacuum Oven Model 280 A, Thermo Fisher Scientific, (Waltham, MA, USA) at 80 °C for 24 h. Then, the dry pellets were cooled to room temperature in a desiccator and weighed. The water-holding capacity was expressed as g of water retained per g of protein.

##### Foaming Capacity

Foaming capacity and foam stability were measured according to the method described by Lin et al. [21]. A mass of 6 g of casein or sodium caseinate was added in 200 mL of distilled water and stirred with a magnetic stirrer overnight. The initial volume of protein solution was measured in a graduated cylinder, poured in a container, and whipped for 6 min with a mixer Oster 2599-033, Newell Brands Canada ULC, (Brampton, ON, Canada) at maximum speed (800 rpm). The whipped solution was immediately transferred into a graduated cylinder, and the volume was measured. The foam capacity (*FC*, in percent) was calculated according to the following equation:

(5) $$FC=\frac{V_{A}-V_{B}}{V_{B}}\times 100$$

where *V_A_* is the foam volume after whipping (in mL), and *V_B_* is the solution volume before whipping (in mL).

##### Foam Stability

To measure foam stability, the foam volume of the solution in the graduated cylinder was recorded after 1, 10, 30, 60, and 120 min. The foam stability (*FS*, in percent) was calculated according to the following equation [21,22]:

(6) $$FS=V_{0}\;x\;\left(\frac{\Delta t}{\Delta V}\right)\times 100$$

where *V*_0_ is the initial foam volume (in mL), Δ*t* is the time interval (in min), and Δ*V* is the change in the volume foam during the time interval (in mL).

##### Emulsifying Activity

Emulsifying activity was measured according to a slightly modified version of the method described by Neto et al. [23]. Protein dispersions of 15 mg/mL (30 mL) were prepared in distilled water. The pH of caseinate solution was adjusted to 7 with NaOH (0.25 M) and the one of casein dispersion was adjusted to 2.5 with HCl (0.5 M); then, 10 mL of the protein dispersion was added in a beaker with 10 mL of canola oil. The solutions were homogenized with an Ultra Turrax T25 basic, IKA-WERKE (Wilmington, NC, USA) at speed 2 (9500 rpm) for 1 min and transferred in 50 mL centrifuge tubes. Then, the emulsions were centrifuged at 1100× *g* for 5 min, and the heights of the total solution and emulsified layer were measured [23,24]. Emulsifying activity (*EA*, in percent) was calculated according to the following equation:

(7) $$EA=\frac{\mathrm{height}\;\mathrm{of}\;\mathrm{emulsified}\;\mathrm{layer}\;}{\mathrm{height}\;\mathrm{of}\;\mathrm{the}\;\mathrm{total}\;\mathrm{solution}\;}\times 100$$

##### Emulsifying Capacity

Emulsifying capacity was measured by the method described by Mohanty et al. [24] with slight modifications. Protein dispersions of 15 mg/mL (10 mL) were prepared in distilled water. The pH of caseinate solution was adjusted as shown previously for the emulsifying activity. Then, 2 mL of the protein solution was transferred in a 50 mL beaker. Canola oil was added gradually (about 5 mL/min) while homogenizing with an Ultra Turrax T25 basic, IKA-WERKE; (Wilmington, NC, USA) at speed 4 (17,500 rpm). The quantity of oil needed for the inversion of emulsion, characterized by a sudden drop in viscosity, was recorded. The emulsifying capacity was expressed as the amount of oil (in g) per 100 mg of protein.

##### Emulsion Stability

Emulsion stability was measured with the method described by Stone and Nickerson [25] with slight modifications. Protein dispersions of 15 mg/mL (10 mL) were prepared in distilled water. The pH of caseinate solution was adjusted as previously for the emulsifying activity. Then, 5.5 mL of the dispersion were added to a 50 mL beaker with 10 mL of canola oil and homogenized with an Ultra Turrax T25 basic, IKA-WERKE; (Wilmington, NC, USA) at speed 2 (9500 rpm) for 2 min. Emulsion was transferred to a 15 mL centrifuge tube. The volume of the aqueous phase was reported after 24 h. The emulsion stability (*ES*, in percent) was calculated according to the following equation:

(8) $$ES=\frac{V_{B}-V_{A}}{V_{B}}\times 100$$

where *V_B_* is the volume of the aqueous phase before emulsification (in mL), *V_A_* is the volume of the aqueous phase after 24 h (in mL).

##### Gelling Capacity

Gelling capacity was determined on sodium caseinate only, according to the method described by Myllarinen et al. [26], with some modifications. Sodium caseinate (6 g) was added in 200 g of distilled water and stirred with a magnetic stirrer overnight at room temperature. The pH of the solution was adjusted to 7 with NaOH (0.25 M). Glucono-delta-lactone was added (0.5% *w*/*w*) to acidify the caseinate solution, and the final solution was vigorously stirred for 2 min. Then, 40 g of the solution was transferred to 50 mL plastic containers, covered with Parafilm, and left for 22 h at room temperature. The gel firmness (in G) was measured with a texturometer TA.XT2; Stable Micro Systems Ltd. (Godalming, UK). The force required to push the tip of an acrylic cylinder of 5 mm diameter into the gel was measured (still in the plastic container) with a speed of 0.5 mm/s.

#### 2.3.7. Statistical Analyses

All treatments and analyses were performed in triplicate for statistical analyses. Data obtained were reported as mean value ± standard deviation. Statistical difference was analyzed by Tukey test (*p* < 0.05) with SigmaPlot software version 14, Systat Software, (San Jose, CA, USA). The composition and functional properties of casein and caseinate from EDBM-UF and chemical processes and from winter and summer milks were subjected to a two-way ANOVA with SigmaPlot software version 14, Systat Software, (San Jose, CA, USA).

## 3. Results and Discussion

### 3.1. Proximal Composition of Milks

Protein and casein contents were higher in winter milk than in summer milk (Table 1). This is in accordance with the literature. Indeed, Bernabucci et al. [27] reported that during summer, heat stress causes a decrease in casein and total protein content. Casein composition is also affected, with a lower concentration in α_s_ and β fractions. This decrease in protein content also influences the content of solids and nonfat solids, which is also lower in summer milk. Urea concentration was higher in summer than in winter milk, which corroborates the literature. Indeed, Godden et al. found a higher amount of urea in summer milk, which would be attributed to the stage of lactation [28].

### 3.2. EDBM Parameters

#### 3.2.1. pH Evolution during Milk Electroacidification

**Figure 2:**
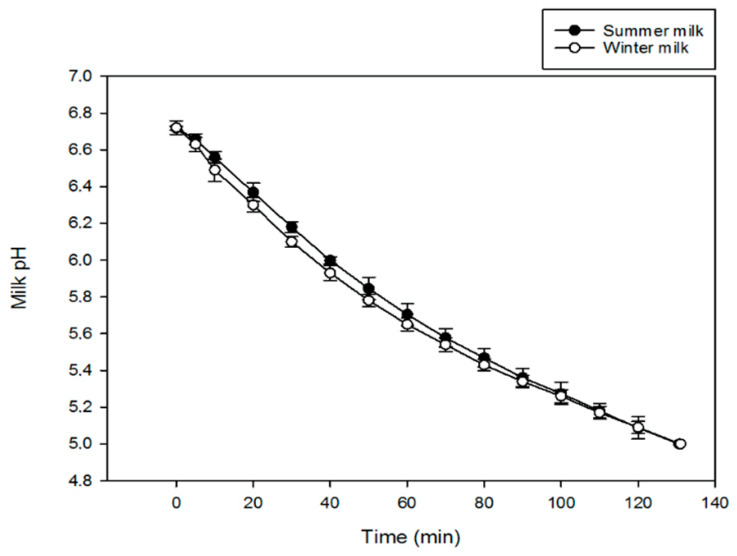
Evolution of milk pH during electrodialysis with bipolar membrane coupled to ultrafiltration (EDBM-UF).

Milk pH was initially around 6.7 and started to gradually decrease due to the addition of H^+^ electrogenerated on the bipolar membrane (Figure 2). The buffering capacity of milk, which is influenced by citrate, phosphate, organic acids, and protein content, caused a slow acidification, which is similar for both seasonal types of milk [29]. The electroacidification duration was also the same for both milks.

**Figure 3:**
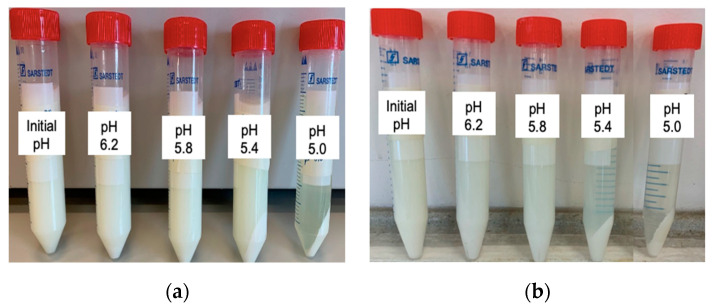
Precipitation kinetics during electroacidification of summer milk (**a**) and winter milk (**b**).

Figure 3a,b show centrifuged samples of milk taken during the EDBM process, at initial pH as well as pH 6.2, 5.8, 5.4, 5.2, and 5.0. Complete precipitation occurred at pH 5.0; however, for the first time, casein precipitation was observed at pH 5.4. This was observed in both milks, but it was more pronounced in winter milk, which had higher casein content (Figure 3b). As stated previously, the conventional precipitation pH of casein during chemical acidification is 4.6. However, in previous studies dealing with the EDBM-UF of skim milk, Masson et al. [9], working with summer milk, found that complete precipitation occurred at pH 4.8, while Mikhaylin et al. [10], working with winter milk, observed a complete precipitation at pH 5.0. This high precipitation pH was obtained due to the reduction in ionic strength during the EDBM-UF process caused by the demineralization of the permeate [30]. However, at pH 5.4, there was still a presence of casein fines in the whey, so the acidification was carried out until complete precipitation, at pH 5.0.

#### 3.2.2. Global System Resistance

**Figure 4:**
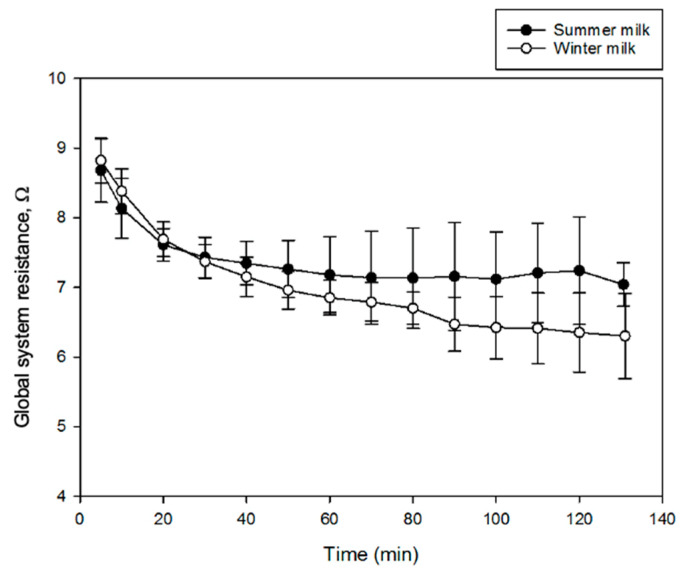
Evolution of global system resistance during electrodialysis with bipolar membrane (EDBM) process of UF permeate of skim milk.

The global system resistance (Figure 4) initially drastically decreased, as highly conductive H^+^ ions were generated by the bipolar membrane [31]. Additionally, the 30 g/L NaCl solution is doubly demineralized. First, Na^+^ ions migrated through the CEMs from compartment 1 toward the basification compartment (Figure 1). Secondly, Cl^−^ ions migrated through the AEM toward compartment 4 [9]. The ultrafiltration permeate was also demineralized. Cations (K^+^, Ca^2+^, Mg^2+^, etc.) migrated across the CEM from compartment 3 to 4 [32]. By the middle of the process, the decrease in resistance was slower and reached a plateau for the summer milk, which may indicate some scaling formation on the cation exchange membranes [33], but the global resistance and the variation in global resistance is really low due to scaling. Compartment 3 limits the global resistance decrease. Indeed, as the process progresses, the UF permeate circulating in this compartment is demineralized while becoming more electroacidified, which slows down the decrease in the global system resistance. Overall, the global system resistance was decreasing during the EDBM process for both types of milk, with a resistance variation (Δ*R*) of 1.8 ± 0.8 Ω for the process using summer milk and 2.5 ± 0.6 Ω for the process using winter milk. Moreover, there was no significant difference between the Δ*R* of processes using milks from both seasons (*p* = 0.066). Finally, the constant resistance (summer milk) and the slight decrease in resistance (winter milk), even toward the end of the process, also indicates that the concentration of the 30 g/L NaCl solution was sufficient and that it was not sufficiently demineralized at the end of the process to affect the system resistance of the EDBM stack [9].

#### 3.2.3. Membranes Characterization

Ion-exchange membranes were characterized by measuring their thickness (mm) and electrical conductivity (mS/cm). These analyses can be used to evaluate the membrane integrity and potential membrane fouling [13]. The membrane thickness of all AEMs, CEMs, and BPMs remained similar before and after three EDBM-UF runs (*p* > 0.05; Table 2). Similarly, regarding electrical conductivity, all CEM and AEM demonstrated no significant difference in conductivity before and after three EDBM runs (*p* > 0.05; Table 2). These results indicate that no fouling or loss of membrane integrity occurred during the EDBM-UF process, which is consistent with the global system resistance results.

### 3.3. Analyses

#### 3.3.1. Casein and Caseinate Composition

Lactose was present in each sample in trace amounts, with a content of less than 0.1% (Table 3). Both casein and caseinate obtained by chemical acidification, from winter and summer milk, had a significantly higher lactose content than ingredients produced by the EDBM-UF process (*p* < 0.05). Generally, the washing process of casein curd influences its lactose content due to the high solubility of lactose. In this study, the same centrifugation steps have been applied for EDBM-UF and chemical ingredients. However, other factors can influence the lactose retention, such as the residual ionic strength of acidification. Indeed, Bazinet et al. [34] have shown that during the washing process of casein, a higher ionic strength results in a reduction in the porosity of the casein coagulum and an increase in its particle size, and therefore, a better retention of lactose. This could explain why the chemical process, which resulted in a higher ionic strength of acidified milk compared to the EDBM-UF process, generates ingredients with a higher lactose content. These results differ from Masson et al. [9], who determined the lactose content of EDBM-UF and chemical casein and caseinate and obtained no significant difference between the ingredients from both processes, and higher values than those obtained in this analysis. This could be due to the different precipitation pH, since they acidified until pH 4.8 with the EDBM-UF process compared to 5.0 in this study. Additionally, this could be attributable to a different composition of milk used since all steps (centrifugation, use of acid generated, etc.) of casein and caseinate preparation were the same as in the present study.

Regarding the ash content, no significant effect was associated to the process, either for casein or caseinate (Table 3). However, the season of milk influenced the ash content of chemical casein, with a lower content in winter, as well as the ash content of EDBM-UF caseinate, with a lower value in summer (*p* < 0.05). There was no interaction between the process and season for both casein and caseinate. These results are somewhat different from those of Masson et al. [9], who also found no difference between caseinate but higher ash content in EDBM-UF casein. The similar ash contents of the ingredients from both processes may indicate that the precipitation pH of EDBM-UF casein of 5.0 compared to 4.6 for chemical casein is not high enough to cause a significant difference in the mineral content of the ingredients. Regarding the seasonal effect, the mineral content of milk is subject to seasonal variations and is affected by several factors, such as lactation stage and animal nutrition [35]. This could explain the difference observed in the ash content of casein. For caseinate, the difference could be attributed to experimental variation in the volume of NaOH added to solubilize casein.

Both casein and caseinate from the EDBM-UF process had significantly higher protein content than from the chemical acidification (*p* < 0.05; Table 3). However, there was neither seasonal effect nor season–process interactions. Casein from the EDBM-UF process in summer and winter had a protein content respectively 8% and 7% higher than chemically produced casein, as well as 13% and 6% higher for summer and winter caseinate. These results corroborate the literature, which reported that casein from electroacidification can reach 95% purity in comparison to 85% purity when it is chemically produced [36]. Bazinet et al. [37] also reported that milk demineralization occurring during electroacidification increased the protein content. However, Masson et al. [9] reported a higher protein content of chemically produced casein and no difference in the protein content of caseinate, which would be related to the different ash content of their ingredients.

#### 3.3.2. Casein and Caseinate Functional Properties

##### Solubility

There was no significant difference between the solubility of caseinate from the chemical and EDBM-UF processes (*p* = 0.702; Table 4) and there was no influence of the milk season (*p* = 0.619; Table 4). Moreover, there was no effect of milk season on caseinate solubility. These results are related to the fact that the solubility of sodium caseinate is strongly affected by the pH of the solution, with a minimum solubility near the isoelectric point and its increase over 90% when the pH is higher than 5.5 [38]. Since the neutralization of casein during caseinate production was performed until pH 6.7 for both chemical and EDBM processes, there was no pH influence on the solubility of obtained caseinates. These results are in accordance with Bazinet et al., showing that the acidification method, chemical or EDBM, had no influence on the solubility of sodium caseinate obtained from fresh and reconstituted milk [34].

##### Hygroscopicity

The hygroscopicity of chemically produced casein was significantly higher than casein from the EDBM-UF process (*p* < 0.05; Table 4). Indeed, chemical caseins from summer and winter milk were 1.2 and 1.1 times more hygroscopic, respectively, than EDBM-UF casein. Caseins from winter milk were more hygroscopic than caseins from summer milk by comparing them from the same process (*p* < 0.05; Table 4). No significant difference was reported between EDBM-UF and chemical caseinate (*p* = 0.306), but both caseinates from summer milk were more hygroscopic (*p* < 0.05; Table 4). However, there was no process and season interaction.

Hygroscopicity describes the tendency of a powder to absorb moisture from its environment. Thus, the higher hygroscopicity of chemical casein can be explained by its higher lactose content, which has a highly hygroscopic behavior [38]. By precipitating at pH 5.0, EDBM-UF casein can retain some colloidal calcium (in a form of Ca_x_H_y_(PO_4_)_z_ bridges) or organic calcium (interacting with phosphoseryl groups of caseins) compared to the complete dissolution of calcium in chemically produced casein at pH 4.6, which can affect the hygroscopicity of casein powders [39]. Additionally, caseinate hygroscopicity in both processes, whatever the milk season, was higher than that of casein, which is consistent with the data reported in the literature. Indeed, the reported hygroscopicity values for sodium caseinate and acid casein were 250 g and 68 g of water per 100 g of solids, respectively [40]. This can be explained by the fact that caseinate hygroscopicity is improved by the resolubilization of casein during neutralization, which leads to the increase in its hydrophilic properties [38].

Since caseins from winter milk should contain more α_S_ and β fractions, which are rich in phosphoseryl groups [27], they were expected to be more hygroscopic. Conversely, the hygroscopicity of casein from both seasons was not significantly different, and caseinates from summer milk were more hygroscopic. These results could be attributable to some differences in the casein fractions present in the final products. Thereby, it would require further analysis on the profile of the ingredients obtained from milk of both seasons in order to discover the mechanisms that affect their hygroscopicity.

##### Water-Holding Capacity

Casein produced by both processes exhibits similar water-holding capacity (*p* = 0.273, Table 4) with higher values for winter milk. However, there was no interaction between process and season. Alternatively to hygroscopicity, the water-holding capacity involves the protein’s ability to trap and retain water in addition to water absorption [41]. According to the literature, acid casein can retain about 2 g of water per g of protein [42], with minimal water-holding capacity occurring between pH 4 and 5.5 and maximum at pH 8 [43]. However, the difference between the pH of chemical and EDBM-UF casein seems to be low to influence their water-holding capacity. Finally, regarding season effect, changes in the casein composition can influence the protein network structure, and eventually, their ability to trap and retain water. Additionally, the higher water-holding capacity of caseins from winter milk can be explained by a higher content in α_S_ and β fractions and their rich amounts of phosphoseryl groups, which can interact with water [27].

##### Foaming Properties

**Figure 5:**
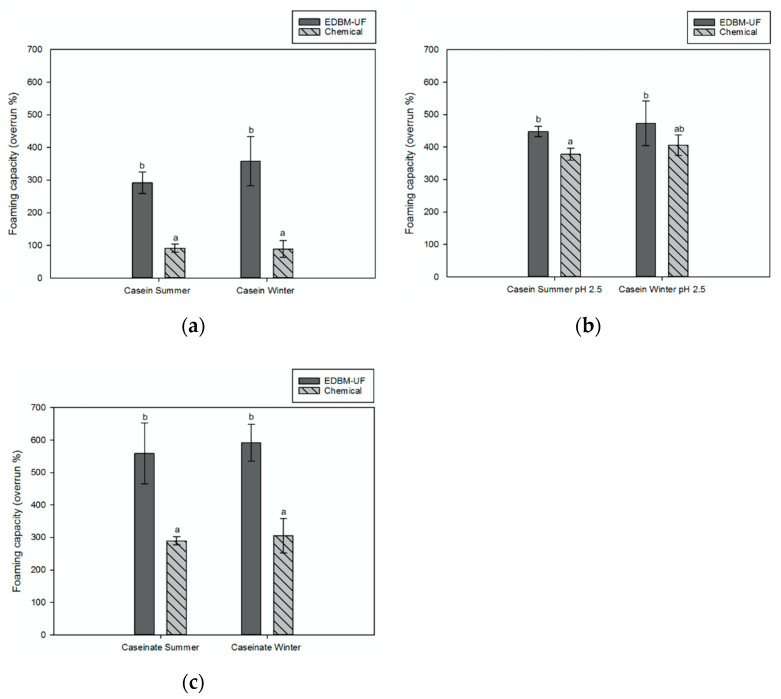
Foaming capacity of (**a**) casein without pH adjustment, (**b**) casein at pH 2.5, and (**c**) caseinate from electrodialysis with bipolar membrane coupled to ultrafiltration (EDBM-UF) and chemical acidification processes for winter and summer milks. The error bar represents SD, and data with different letters are significantly different at *p* < 0.05.

All EDBM-UF ingredients from both winter and summer milks had significantly higher foaming capacity than chemical ones (*p* < 0.05; Figure 5). Indeed, EDBM-UF caseins without pH adjustment demonstrated 3.2 and 4.0 times higher foaming capacity than chemical ones for summer and winter seasons, respectively. Additionally, EDBM-UF caseins at pH 2.5 had 1.2 times higher foaming capacity for both studied milks. Finally, EDBM-UF caseinate had 1.9 times higher foaming capacity compared to the chemical one for both milk seasons.

**Figure 6:**
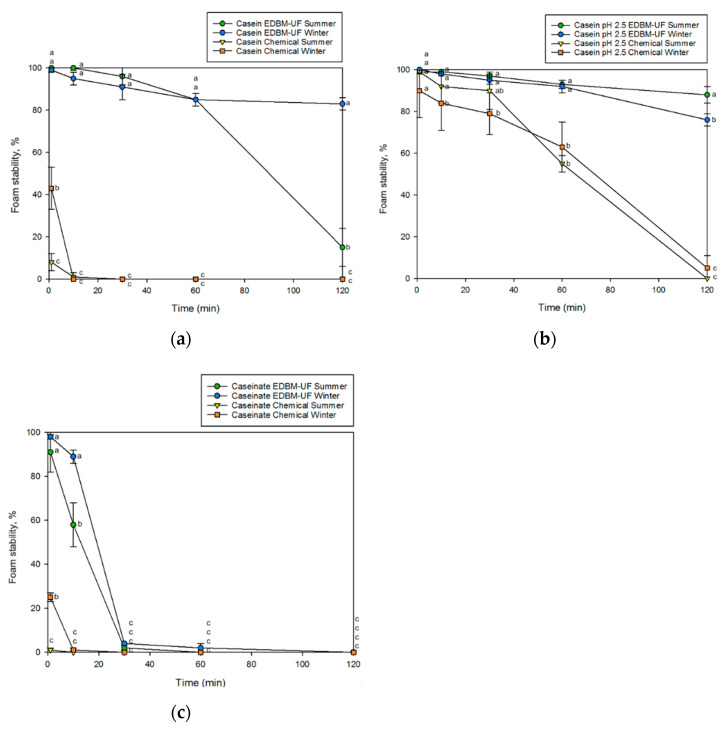
Foam stability of (**a**) casein without pH adjustment, (**b**) casein at pH 2.5, and (**c**) caseinate from electrodialysis with a bipolar membrane coupled to ultrafiltration (EDBM-UF) and chemical acidification processes for winter and summer milks. The error bar represents SD, and data with different letters are significantly different at *p* < 0.05.

A similar tendency to foaming capacity was observed for foam stability. Indeed, the foam of casein without pH adjustment produced by the EDBM-UF process was significantly more stable during two hours compared to chemically produced casein (*p* < 0.05; Figure 6a). Otherwise, the foam of casein at pH 2.5 from both processes was equivalently stable in the first minutes, but the foam of casein produced by EDBM-UF was significantly more stable after 30 min in winter and 60 min in summer (*p* < 0.05; Figure 6b). Finally, the foam of caseinate produced by the EDBM-UF process was significantly more stable during 10 min (*p* < 0.05; Figure 6c). Except for casein produced by EDBM-UF, where the foam stability of winter casein was still high after 120 min (more than 80% vs. 18%), the season of milk had little or no impact on the foam stability.

The foaming properties of casein depend on several factors. First, their solubility and presence of charged or polar groups dictate their ability and rapidity to diffuse at the water-air interface and reduce the interfacial tension, which is the first step in foam formation [44]. Depending on the pH, the charges and surface activity of casein are modified. Indeed, the foaming capacity of casein is better when pH is far from its isoelectric point (pI) because of a better solubility, flexibility, and a denser adsorption layer [45,46]. Indeed, near the pI, the structure of casein is more compact, and protein–protein interactions are favored. Thus, proteins are less available to adsorb at the water–air interface and to reduce the interfacial tension [40,44,46]. This could explain why caseinate and casein at pH 2.5 exhibit higher foaming capacities than casein without pH adjustment. Finally, another important factor in foam properties is protein concentration. Indeed, according to the literature, an increase in protein concentration causes a better foam volume augmentation and foam stability. On the one hand, higher protein concentrations cause a faster rate of adsorption and therefore a greater surface activity. On the other hand, the thickness of the protein film formed at the water–air interface is increased, which allows a better foam stability [46,47]. Thus, EDBM-UF ingredients have higher protein content, which can explain their higher foaming properties.

Bazinet et al. [37] have compared the foaming properties of casein from reconstituted milk produced by EDBM at different ionic strengths with a final precipitation pH of 4.6 and chemical-acidified casein. They reported no significant difference for both processes. Since precipitation was performed at the same pH for all the ingredients, this could explain why they did not notice any difference.

##### Emulsifying Properties

Emulsifying properties were similar for all ingredients (*p* > 0.05; Table 4), indicating that there was no significant impact of the acidification process nor milk season. In fact, the amphiphilic character of caseins gives them important surface-active properties. They can adsorb at the water-oil interface, with hydrophobic regions interacting with the oily phase and hydrophilic regions interacting with the aqueous phase. The emulsifying properties are highly influenced by the pH of the solution, which modifies the protein charges and solubility [43]. Since the pH of the protein solutions was adjusted prior to emulsifying properties analyses, the hydrophobicity and solubility of the ingredients from both processes should be similar. Hence, the surface-active properties are expected to be similar, and no difference in *EC* and *EA* should be reported. Moreover, regarding *ES,* the electrostatic repulsions and steric hindrance are the main factors [48,49]. Therefore, it is not surprising to obtain similar results, since the electrical charges covering the surface of the fat droplets are expected to be similar as well as the size and conformation of casein’s side chains. Finally, protein concentration in the aqueous phase generally influences the emulsifying properties, and as demonstrated previously, EDBM-UF ingredients had a higher protein content. However, the concentration of powder used in those analyses is relatively low in comparison to the concentration used in foaming properties analyses, and the difference in protein content may not be enough to cause a significant difference in the emulsifying properties. In addition, since the emulsifying capacity is expressed as g of oil per 100 mg of dry proteins, this potential effect is eliminated.

Bazinet et al. [34] compared the emulsifying stability of fresh milk chemically and electrochemically (EDBM) acidified. The authors observed no significant difference between chemical and electrochemical acidified milk, which corroborates the results of the current study.

##### Gelling Capacity

No significant difference was observed between the gelling capacities of caseinate from chemical and EDBM-UF processes (*p* = 0.518; Table 4) and between seasons of the milk (*p* = 0.092; Table 3). The gelation of casein is caused by the acidification of GDL, which neutralizes the negative charges of casein and causes their aggregation, which is followed by the formation of a three-dimensional network. Under pH 5.0, the reduction in electrostatic repulsions allows an increase in hydrophobic interactions and the beginning of gelation. Maximum firmness occurs at pH 4.6 [50]. Since pH was adjusted to 7.0 prior to acidification and the same GDL concentration was used as well as other experimental parameters that influence gelation properties of casein, such as temperature and ionic strength, the firmness of the gels formed by both caseinates should be equivalent.

## 4. Conclusions

This study analyzed for the first time the functional properties of casein and caseinate produced by the hybrid EDBM-UF process. The impact of the season of milk has also been studied. The results showed that most of the functional properties of EDBM-UF ingredients were equivalent to those produced by conventional chemical acidification/basification; some of them were even superior. Indeed, because of the higher protein content and higher precipitation pH, the foaming properties and hygroscopicity of the EDBM-UF ingredients were improved. Indeed, EDBM-UF caseins without pH adjustment demonstrated 3.2 times and 4.0 times higher foaming capacity than chemical one for summer and winter seasons, respectively, while EDBM-UF caseins at pH 2.5 had 1.2 times higher foaming capacity for both studied milks, and EDBM-UF caseinate had 1.9 times higher foaming capacity compared to the chemical one for both milk seasons. Moreover, chemical casein from summer and winter milk types was 1.2 times and 1.1 times more hygroscopic, respectively, than EDBM-UF casein. Regarding the impact of the seasons, it has been shown that winter milk provides casein with better water retention and lower hygroscopicity compared to summer milk. Furthermore, in addition to being eco-friendlier than the chemical process, the fact that EDBM-UF generates ingredients with a higher protein content and better functionality makes this process even more eco-efficient than that demonstrated by the life cycle assessment of Mikhaylin et al. [8]. Therefore, the EDBM-UF process could represent an interesting method to produce casein and caseinate as an alternative to chemical acidification. The next steps would be to perform a further analysis on the profile of the ingredients obtained by both processes and seasons in order to discover the mechanisms that affect their functional properties, and the scale-up of the process for industrial uses.

## Figures and Tables

**Table 1:** Skim milk composition.

Compound	Summer Milk	Winter Milk
Fat (% m/m)	0.07 ± 0.01 ^a^*	0.07 ± 0.01 ^a^
Protein (% m/m)	3.29 ± 0.01 ^a^	3.42 ± 0.03 ^b^
Lactose (% m/m)	4.74 ± 0.01 ^a^	4.74 ± 0.02 ^a^
Solids (% m/m)	9.02 ± 0.01 ^a^	9.16 ± 0.03 ^b^
Nonfat solids (% m/m)	8.26 ± 0.01 ^a^	8.40 ± 0.04 ^b^
Casein (g/L)	26.37 ± 0.05 ^a^	27.47 ± 0.19 ^b^
NPN/CU * (mg/100 g)	13.20 ± 0.80 ^b^	11.50 ± 0.80 ^a^

* Data on a line with different letters (a, b) are significantly different at *p* < 0.05. NPN/CU refers to Non-protein nitrogen/Calculated urea.

**Table 2:** Membranes characterization.

Membrane	Thickness (mm)		Conductivity (mS/cm)	
	Before	After	Before	After
AEM	0.142 ± 0.004 ^a^*	0.142 ± 0.005 ^a^	5.4 ± 0.4 ^a^	5.3 ± 0.2 ^a^
CEM1	0.151 ± 0.002 ^a^	0.151 ± 0.001 ^a^	8.8 ± 0.6 ^a^	7.9 ± 0.7 ^a^
CEM2	0.152 ± 0.001 ^a^	0.153 ± 0.001 ^a^	8.6 ± 0.3 ^a^	7.9 ± 0.5 ^a^
CEM3	0.152 ± 0.001 ^a^	0.153 ± 0.002 ^a^	8.9 ± 0.2 ^a^	8.6 ± 0.6 ^a^
BPM	0.240 ± 0.003 ^a^	0.241 ± 0.003 ^a^	-	-

* Data on a line with different letters (a) are significantly different at *p* < 0.05.

**Table 3:** Impact of process and seasonal variations on casein and caseinate composition.

Compound	Casein				Caseinate			
	EDBM-UF		Chemical		EDBM-UF		Chemical	
	Summer	Winter	Summer	Winter	Summer	Winter	Summer	Winter
Lactose (% dry weight)	0.037 ± 0.003 ^a^*	0.038 ± 0.005 ^a^	0.078 ± 0.016 ^b^	0.089 ± 0.010 ^b^	0.028 ± 0.006 ^a^	0.038 ± 0.008 ^a^	0.068 ± 0.006 ^b^	0.087 ± 0.010 ^b^
Ash (% dry weight)	3.18 ± 0.13 ^b^	2.91 ± 0.23 ^ab^	3.20 ± 0.10 ^b^	2.81 ± 0.02 ^a^	5.07 ± 0.50 ^a^	5.77 ± 0.03 ^b^	5.42 ± 0.32 ^ab^	5.72 ± 0.05 ^b^
Protein (% dry weight)	92 ± 2 ^b^	93 ± 1 ^b^	85 ± 4 ^a^	87 ± 2 ^a^	88 ± 1 ^b^	89 ± 1 ^b^	79 ± 5 ^a^	83 ± 3 ^a^

* Data on a line with different letters (a, b) are significantly different at *p* < 0.05. Caseins are compared with caseins and caseinates are compared with caseinates.

**Table 4:** Functional properties of casein and caseinate.

Functional Property	Casein				Caseinate			
	EDBM-UF		Chemical		EDBM-UF		Chemical	
	Summer	Winter	Summer	Winter	Summer	Winter	Summer	Winter
Solubility (%)	-	-	-	-	98.3 ± 0.7 ^a^*	98.1 ± 0.5 ^a^	98.2 ± 0.9 ^a^	98.8 ± 1.3 ^a^
Water-holding capacity (g of water/g of protein)	2.01 ± 0.05 ^a^	3.09 ± 0.14 ^b^	2.10 ± 0.21 ^a^	3.33 ± 0.43 ^b^	-	-	-	-
Hygroscopicity (g of water/100 g of dry solids)	24.9 ± 0.7 ^b^	22.7 ± 0.8 ^a^	30.0 ± 0.7 ^c^	24.4 ± 0.1 ^b^	41.1 ± 3.4 ^b^	32.1 ± 2.1 ^a^	44.6 ± 3.6 ^b^	32.2 ± 1.9 ^a^
Emulsifying activity (%)	48 ± 2 ^a^	48 ± 1 ^a^	47 ± 2 ^a^	49 ± 1 ^a^	46 ± 2 ^a^	49 ± 1 ^a^	47 ± 2 ^a^	47 ± 3 ^a^
Emulsion stability (%)	48 ± 2 ^a^	49 ± 5 ^a^	47 ± 1 ^a^	43 ± 3 ^a^	36 ± 2 ^a^	37 ± 4 ^a^	37 ± 2 ^a^	37 ± 3 ^a^
Emulsifying capacity (g of oil/100 g of dry proteins)	33 ± 3 ^a^	32 ± 2 ^a^	34 ± 2 ^a^	33 ± 2 ^a^	34 ± 1 ^a^	38 ± 4 ^a^	35 ± 2 ^a^	37 ± 2 ^a^
Gel firmness (G)	-	-	-	-	22 ± 5 ^a^	26 ± 4 ^a^	22 ± 1 ^a^	24 ± 1 ^a^

* Data on a line with different letters (a, b, c) are significantly different at *p* < 0.05. Caseins are compared with caseins, and caseinates are compared with caseinates.

## Data Availability

The data presented in this study are available on request from the corresponding author.

## References

[1] Wong D.W.S., Camirand W.M., Pavlath A.E., Parris D.N., Friedman D.M. (1996). Structures and functionalities of milk proteins, Critical Reviews in Food Science & Nutrition. Crit. Rev. Food Sci. Nutr..

[2] Chandan R.C., Kilara A. (2011). Dairy Ingredients for Food Processing.

[3] Gaudichon C. (2001). Les Protéines Laitières: Intérêts Technologiques et Nutritionnels.

[4] Paul M. (2007). Formation of Bioactive Peptides from Dairy Products.

[5] Sarode A., Sawale P., Khedkar C., Kalyankar S., Babalerro B., Finglas P., Toldra F. (2016). Casein and Caseinate: Methods of Manufacture. The Encyclopedia of Food and Health.

[6] Huppertz T., Fox P.F., Kelly A.L., Yada R.Y. (2018). The caseins: Structure, stability, and functionality. Proteins in Food Processing.

[7] Brule G., Fauquant J., Maubois J.-L. (1979). Preparation of “Native” Phosphocaseinate by Combining Membrane Ultrafiltration and Ultracentrifugation. J. Dairy Sci..

[8] Mikhaylin S., Patouillard L., Margni M., Bazinet L. (2018). Milk protein production by a more environmentally sustainable process: Bipolar membrane electrodialysis coupled with ultrafiltration. Green Chem..

[9] Masson F.-A., Mikhaylin S., Bazinet L. (2018). Production of calcium- and magnesium-enriched caseins and caseinates by an ecofriendly technology. J. Dairy Sci..

[10] Mikhaylin S., Sion A.-V. (2016). Improvement of a sustainable hybrid technology for caseins isoelectric precipitation (electrodialysis with bipolar membrane/ultrafiltration) by mitigation of scaling on cation-exchange membrane. Innov. Food Sci. Emerg. Technol..

[11] Carr A., Golding M. (2016). Functional Milk Proteins Production and Utilization: Casein-Based Ingredients. Adv. Dairy Chem..

[12] AOAC (1995). Method 972.16 Fat, Lactose, Protein, and Solids in Milk. Official Methods of Analysis of AOAC International.

[13] Lemay N., Mikhaylin S., Bazinet L. (2019). Voltage spike and electroconvective vortices generation during electrodialysis under pulsed electric field: Impact on demineralization process efficiency and energy consumption. Innov. Food Sci. Emerg. Technol..

[14] (2007). Milk and Milk Products-Determining of Lactose Content by High-Performance Liquid Chromatography (Reference Method).

[15] FAO Nitrogen and Protein Content Measurement and Nitrogen to Protein Conversion Factors for Dairy and Soy Protein-Based Foods: A Systematic Review and Modelling Analysis. https://apps.who.int/iris/bitstream/handle/10665/331206/9789241516983-eng.pdf.

[16] AOAC (1995). Method 945.46: Ash in milk. Official Methods of Analysis of AOAC International.

[17] Haque E., Whittaker A.K., Gidley M.J., Deeth H.C., Fibrianto K., Bhandari B.R. (2012). Kinetics of enthalpy relaxation of milk protein concentrate powder upon ageing and its effect on solubility. Food Chem..

[18] Anema S.G., Pinder D.N., Hunter R.J., Hemar Y. (2006). Effects of storage temperature on the solubility of milk protein concentrate (MPC85). Food Hydrocoll..

[19] Ma J.-J., Mao X.-Y., Wang Q., Yang S., Zhang D., Chen S.-W., Li Y.-H. (2014). Effect of spray drying and freeze drying on the immunomodulatory activity, bitter taste and hygroscopicity of hydrolysate derived from whey protein concentrate. Food Sci. Technol..

[20] Mirmoghtadaie L., Kadivar M., Shahedi M. (2009). Effects of succinylation and deamidation on functional properties of oat protein isolate. Food Chem..

[21] Lin M.J.Y., Humbert E.S., Sosulski F.W. (1974). Certain functional properties of sunflower meal products. J. Food Sci..

[22] Kato A., Takahashi A., Matsudomi N., Kobayashi K. (1983). Determination of Foaming Properties of Proteins by Conductivity Measurements. J. Food Sci..

[23] Neto V.Q., Narain N., Silva J.B., Bora P.S. (2001). Functional properties of raw and heat processed cashew nut (*Anacardium occidentale*, L.) kernel protein isolates. Mol. Nutr..

[24] Mohanty B., Mulvihill D.M., Fox P.F. (1988). Emulsifying and Foaming Properties of Acidic Caseins and Sodium Caseinate. Food Chem..

[25] Stone A.K., Nickerson M.T. (2012). Formation and functionality of whey protein isolate-(kappa-, iota-, and lambda-type) carrageenan electrostatic complexes. Food Hydrocoll..

[26] Myllarinen P., Buchert J., Autio K. (2005). Effect of transglutaminase on rheological properties and microstructure of chemically acidified sodium caseinate gels. Int. Dairy J..

[27] Bernabucci U., Basiricò L., Morera P., Dipasquale D., Vitali A., Cappelli F.P., Calamar L. (2015). Effect of summer season on milk protein fractions in Holstein cows. J. Dairy Sci..

[28] Godden S.M., Lissemore K.D., Kelton D.F., Lesli K.E., Walton J.S., Lumsden J.H. (2001). Factors Associated with Milk Urea Concentrations in Ontario Dairy Cows. J. Dairy Sci..

[29] Salaün F., Mietton B., Gaucheron F. (2005). Buffering capacity of dairy products. Int. Dairy J..

[30] Bazinet L., Lamarche F., Ippersiel D., Gendron C., Mahdavi B., Amiot J. (2000). Comparaison of Electrochemical and Chemical Acidification of skim milk. J. Food Sci..

[31] Bazinet L., Lamarche F., Ippersiel D., Amiot J. (1999). Bipolar Membrane Electroacidification To Produce Bovine Milk Casein Isolate. J. Agric. Food Chem..

[32] Bazinet L., Ippersiel D., Gendron C., Beaudry J., Mahdavi B., Amiot J., Lamarche F. (2000). Cationic balance in skim milk during bipolar membrane electroacidification. J. Membr. Sci..

[33] Mikhaylin S., Nikonenko V., Pourcelly G., Bazinet L. (2016). Hybrid bipolar membrane electrodialysis/ultrafiltration technology assisted by a pulsed electric field for casein production. Green Chem..

[34] Bazinet L., Ippersiel D., Gendron C., Tétreault C., René-Paradis J., Beaudry J., Britten M., Mahdavi B., Amiot J., Lamarche F. (2002). Comparison between reconstituted and fresh skim milk chemical and electrochemical acidifications. J. Sci. Food Agric..

[35] Li S., Ye A., Singh H. (2019). Seasonal variations in composition, properties, and heat-induced changes in bovine milk in a seasonal calving system. J. Dairy Sci..

[36] Mier M.P., Ibañez R., Ortiz I. (2008). Influence of process variables on the production of bovine milk casein by electrodialysis with bipolar membranes. Biophys. Eng. J..

[37] Bazinet L., Gendron C., Ippersiel D., René-Paradis J., Tétreault C., Beaudry J., Britten M., Mahdavi B., Amiot J., Lamarche F. (2002). Effects of Type of Added Salt and Ionic Strength on Physicochemical and Functional Properties of Casein Isolates Produced by Electroacidification. J. Agric. Food Chem..

[38] Zayas J.F. (1997). Functionality of Proteins in Food.

[39] Le Graet Y., Brulé G. (1993). Les équilibres minéraux du lait: Influence du pH et de la force ionique. Lait.

[40] Kinsella J.E., Morr C.V. (1984). Milk proteins: Physicochemical and functional properties. Crit. Rev. Food Sci. Nutr..

[41] Kneifel W., Seiler A. (1993). Water-holding Properties of Milk Protein Products—A Review. Food Struct..

[42] Teo C.T., Munro P.A., Singh H., Hudson R.C. (2009). Effects of pH and temperature on the water-holding capacity of casein curds and whey protein gels. J. Dairy Res..

[43] Cayot P., Lorient D. (1998). Structures et Technofonctions des Protéines de Lait.

[44] Lorient D., Closs B., Courthaudon J.-L. (1989). Surface properties of the bovine casein components: Relationships between structure and foaming properties. J. Dairy Sci..

[45] Sarkar A., Singh H., McSweeney P., O’Mahony J. (2016). Emulsions and Foams Stabilized by Milk Proteins. Volume 1B: Proteins: Applied Aspects.

[46] Marinova K.G., Basheva E.S., Nenova B., Temelska M., Mirarefi A.Y., Campbell B., Ivanov I.B. (2009). Physico-chemical factors controlling the foamability and foam stability of milk proteins: Sodium caseinate and whey protein concentrates. Food Hydrocoll..

[47] Kinsella J.E. (1981). Functional properties of proteins: Possible relationships between structure and function in foams. Food Chem..

[48] Leman J., Kinsella J.E., Kilara A. (1989). Surface activity, film formation, and emulsifying properties of milk proteins. Crit. Rev. Food Sci. Nutr..

[49] Singh H., Ye A., Thompson A., Boland M., Singh H. (2009). Interactions and functionality of milk proteins in food emulsions. Milk Proteins from Expression to Food.

[50] Lucey J.A., Fox P.F., Sweeney P.L.H.M., Cogan T.M., Guinee T.P. (2004). Formation, Structural Properties, and Rheology of Acid-Coagulated Milk Gels. Cheese, Chemistry, Physics and Microbiology.

